# Supplementary figures and images for: B Cell Receptor’s function in virus entry: Anti-SARS-CoV-2 B cell receptors can mediate viral entry in an ACE2-independent mechanism
Source: PLoS Pathog. 2026 Feb 6;22(2):e1013946. doi: 10.1371/journal.ppat.1013946 (PMC12893656; doi:10.1371/journal.ppat.1013946)

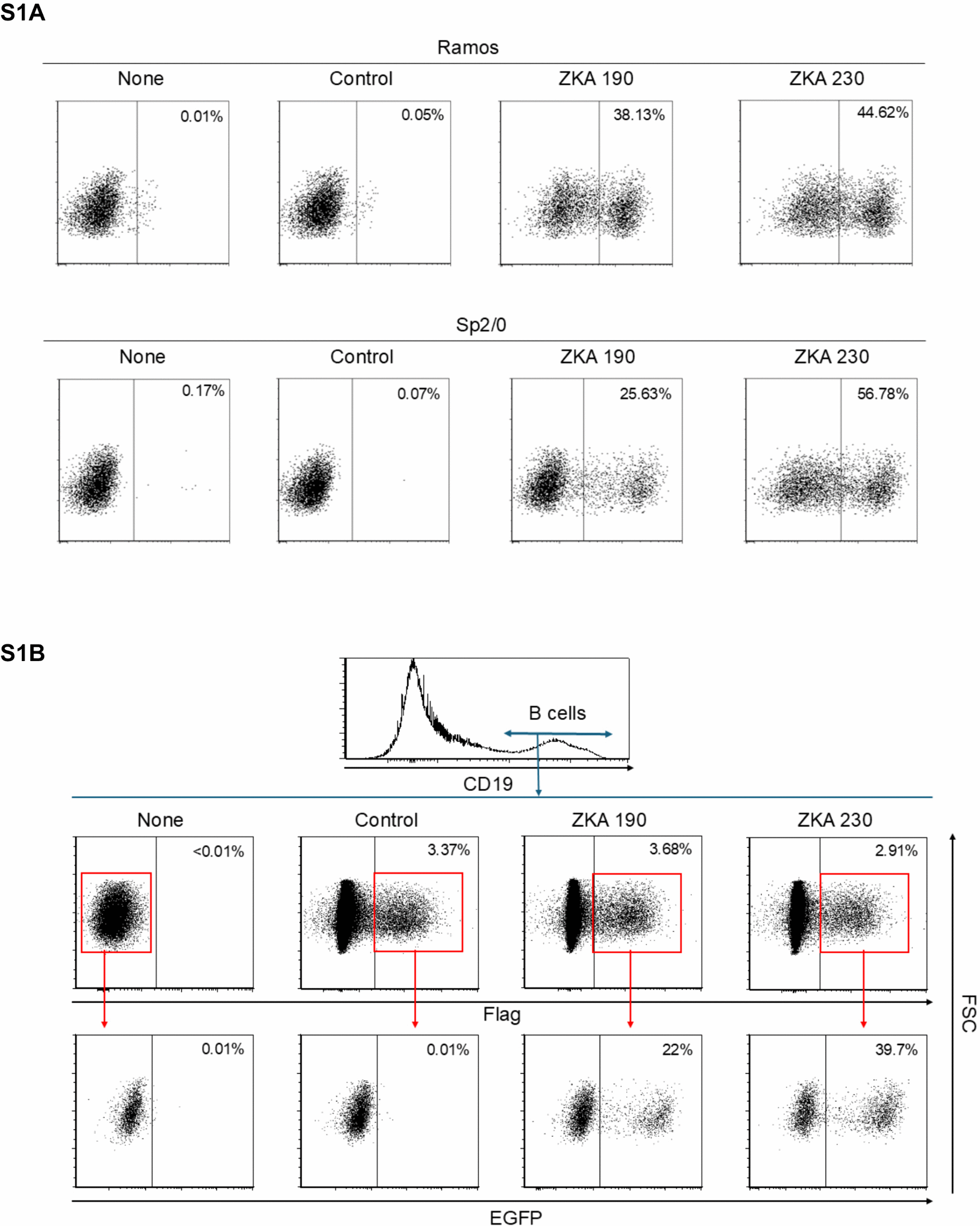

Supplement: S1 Fig — A) Representative flow cytometric profiles of cells stained intracellularly with an Alexa Fluor 647-conjugated anti-ZIKV E protein antibody following ZIKV infection. B) Representative flow cytometric profiles illustrating the gating strategy for analyzing in vitro ZIKV infection of primary human B cells expressing either control or anti-ZIKV BCRs. CD19 + events were gated to identify the B cell population, with subsequent gating for PE-conjugated anti-Flag staining to identify BCR-expressing cells. Both untransduced and BCR-expressing B cells were then assessed for ZIKV replicon infection via EGFP reporter expression. (TIF) [file ppat.1013946.s001.tif]

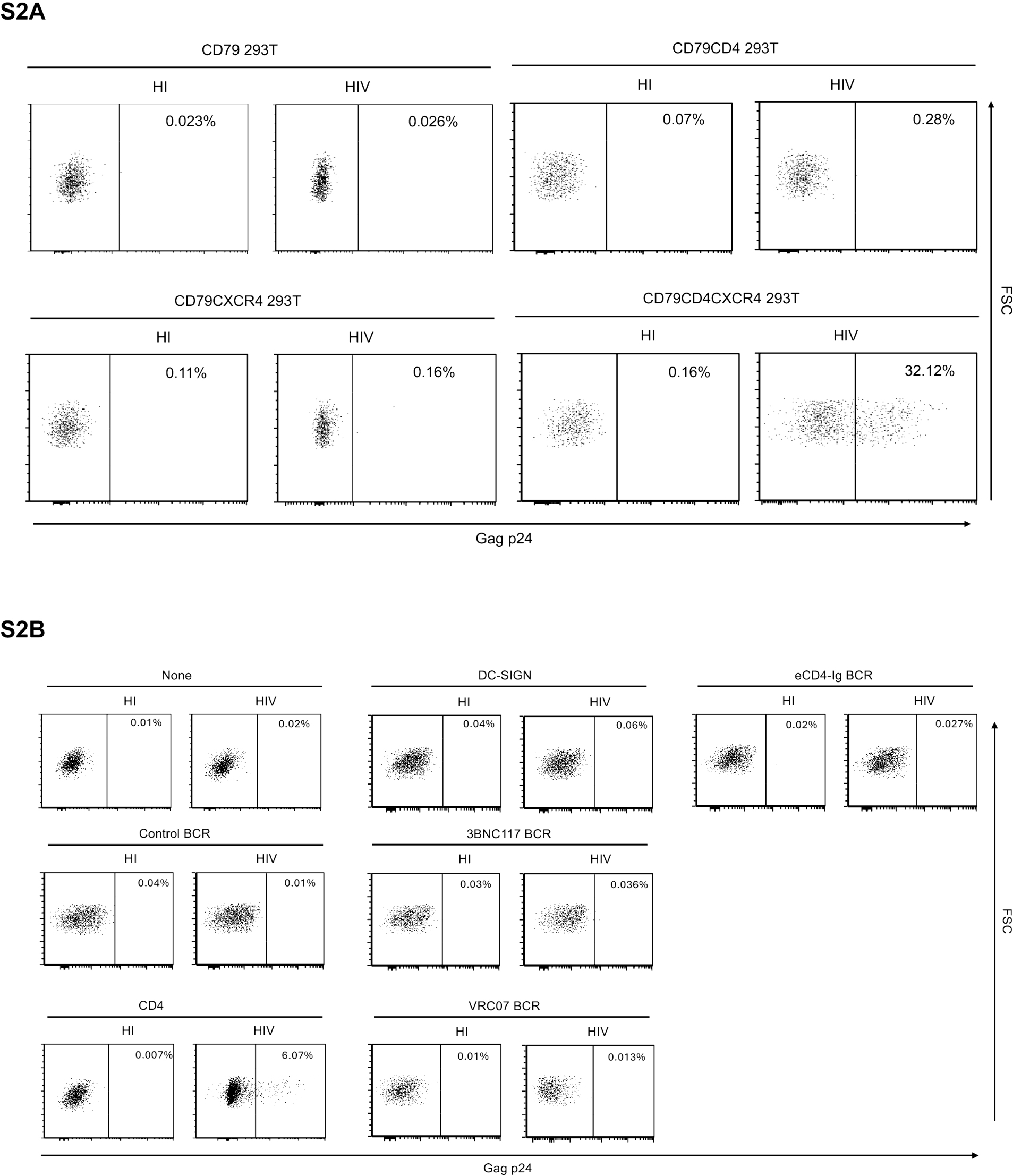

Supplement: S2 Fig — A) Representative flow cytometric profiles of intracellular HIV-1 Gag p24 expression in CD79 293T or CD79CXCR4 293T cells, with or without ectopic CD4 expression, following infection with either heat-inactivated or infectious HIV-1 NL4-3. B) Representative flow cytometric profiles of intracellular HIV-1 Gag p24 expression in CD79 CXCR4 cells with or without ectopic expression of DC-SIGN, control BCR, or anti-HIV-1 BCR derived from 3BNC117, VRC07, and eCD4-Ig, and CD79 CD4CXCR4 293T cells (shown as CD4) following infection with either heat-inactivated or infectious HIV-1 NL4-3. (TIF) [file ppat.1013946.s002.tif]

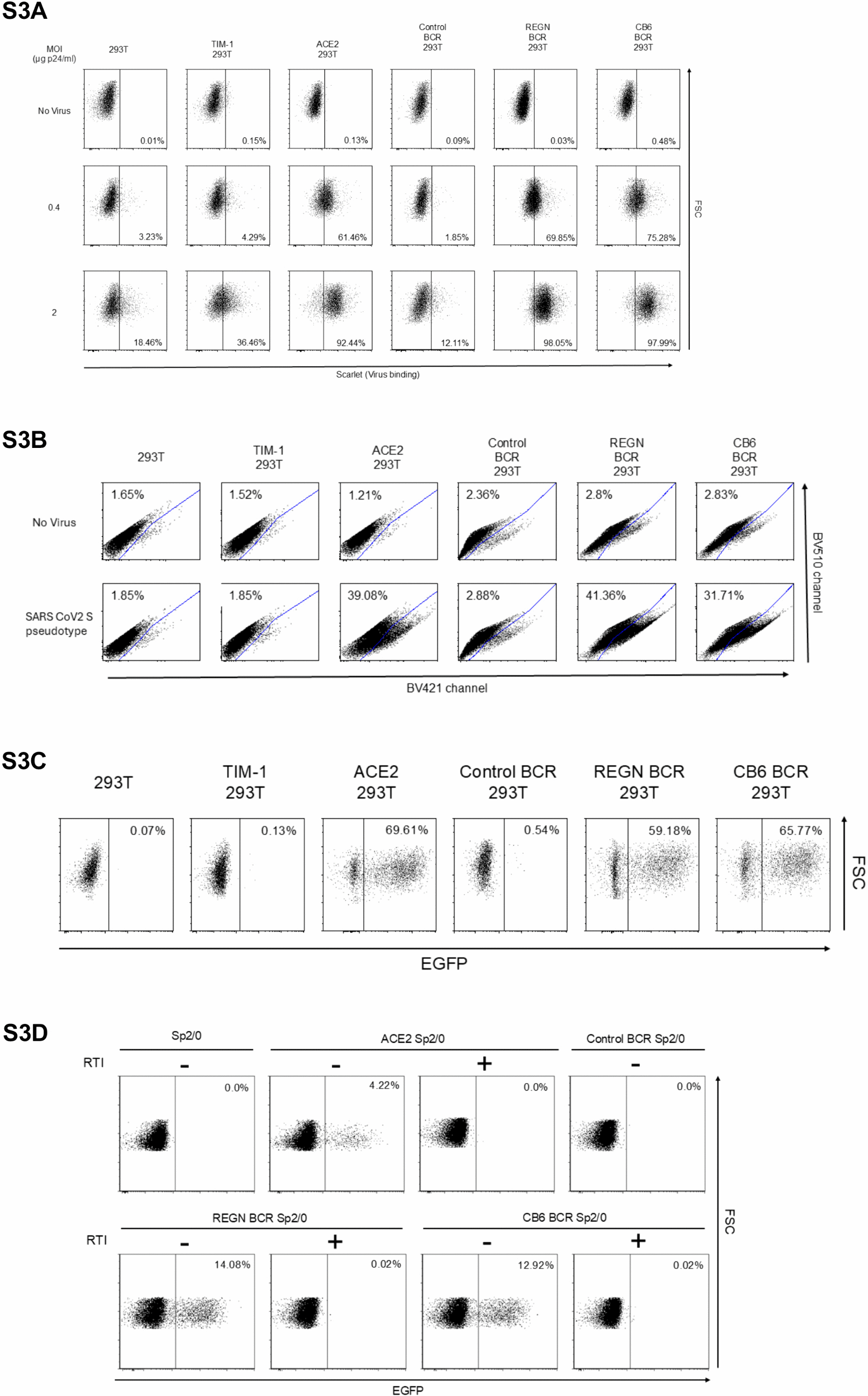

Supplement: S3 Fig — A) Flow cytometric profiles of S protein pseudotyped lentiviral vector binding to cells ectopically expressing TIM-1, ACE2, or BCRs [control or anti-SARS-CoV-2 (REGN and CB6)]. B) Representative flow cytometric profiles of virus fusion/entry assays detected by the elimination of FRET induced by cleavage of CCF4-AM with virion-incorporated β-lactamase. C) Representative flow cytometric profiles of transduction of CD79 Sp2/0 cells ectopically expressing TIM-1, ACE2, or BCRs [control or anti-SARS-CoV-2 (REGN and CB6)] with S protein pseudotyped lentiviral vector expressing EGFP transgene. D) Representative flow cytometric profiles of CD79 Sp2/0 cells ectopically expressing either ACE2 or BCRs (control or anti-SARS-CoV-2 [REGN and CB6]) following transduction with an S protein-pseudotyped lentiviral vector encoding an EGFP transgene, conducted in the presence or absence of the reverse transcriptase inhibitor (RTI) Nevirapine. (TIF) [file ppat.1013946.s003.tif]
