# Supplementary material for: B Cell Receptor’s function in virus entry: Anti-SARS-CoV-2 B cell receptors can mediate viral entry in an ACE2-independent mechanism
Source: PLoS Pathog. 2026 Feb 6;22(2):e1013946. doi: 10.1371/journal.ppat.1013946 (PMC12893656; doi:10.1371/journal.ppat.1013946)
Supplement: Attachment — Submitted filename: Larios et al PPATHOGENS-D-25-02468.docx [file ppat.1013946.s005.docx]

The manuscript by Larios et al, explores the hypothesis that antiviral B cell receptors (BCRs) can mediate the processes of viral entry, fusion, or infection in the absence of canonical host receptors. The authors report that anti-ZIKV BCRs in heterologous systems mediate binding and infection of ZIKV, anti-HIV-1 BCRs mediate viral attachment without enabling fusion or entry, whereas anti-SARS-CoV-2 Spike protein BCRs purportedly facilitate both attachment, fusion, entry, and infection independently of ACE2. However, the reliance on extensive overexpression in vitro systems and selective conditions raises concerns about the physiological relevance and reproducibility of these findings. The conclusions, though framed as revealing a novel entry pathway with immunological implications, rest on a narrow experimental foundation that does not convincingly support the broader claims and are not reflective of the pathophysiology of these viruses.

Major issues:

Across the different experimental systems there are several variables for each and this lends itself to overreliance on blanket terms like “infection” , “attachment”, “fusion”. These terms are not clearly enough defined in the legends or on the graphs in figures, prompting a deep dive in the methods to understand the experimental setup. For example, how is infection defined in Fig 1B,C? This is unclear in results text or legend. The methods (lines 469-470) mention staining for flavivirus group antigen (is this 4G2?) in permeabilized cells, so this could just be viral uptake rather than infection.

MOIs should be given where applicable (e.g. ZIKV RVP experiments, SARS2 infection)

Fig 1D: The presented data raise questions about the experimental system that is critical for claiming that ZIKV can infect primary human B cells. For the red line, how can there be that much IgG1 staining in FLAG-negative cells from a total B cell population (assuming CD19 gating was done here) that is >80% endogenously IgM/IgD-positive? There should only be a small fraction of endogenous IgG1+ cells (~ 1 - 10%) in total B cells. What fraction of B cells are transduced? Also, throughout please state clearly in legend what the control BCR is.

Figure 2A: Looking at very small effects. Even in IFNARKO mice, there is limited transduction of BCRs (percentages should be given) and of those transduced cells, only a small fraction of these become GFP+ (3-6%) following in vivo ZIKV replicon administration via i.v. injection. Though a t-test indicates statistical significance, the physiological relevance is unclear.

In Figures 4 and 5 the message is that SARS2-specific BCRs facilitate binding and fusion of SARS2 pseudotyped lentivirus and infection with SARS2 in 293T cells to the same extent as ACE2. Furthermore, cathepsin L activity is similarly required for ACE2 or BCR-mediated infection. The labels A,B,C in Fig 5A,B are not clearly indicated so difficult to confirm what is being tested. On line 206 the authors cite refs 14, and 43 (which is a duplicate of ref 13) as evidence that SARS-CoV-2 infects B cells in vivo. Ref 14 is an atlas of SARS2 RNA-containing lung cells during COVID-19 wherein phagocytic cells in general have SARS2 RNA, with B/plasma cells having a weak representation. Ref 13/43 relies mainly on dsRNA staining (specific for SARS2?, not described) and SARS2 antigen detected with serum from COVID-19 patient. So these references are not convincing for the rationale. Furthermore, SARS2-specific BCR-bearing memory B cells which expand following exposure or vaccination could potentially mediate increasing viral load and disease following SARS2 challenge, but this is not borne out, like what happens for dengue.

Other issues:

Introduction:

Line 117: dengue virus doesn’t “induce conformational changes”, rather the lowering of pH upon endocytosis does.

Line 120: cannot say “universally” based on one other manuscript.

Line 126: what is meant by “facilitate”? rather in the Influenza paper, BCR signaling sensitizes cell for infection and for RSV, the infection is of a rather narrow subset of B cell and is through CX3CR1.

Figures:

Fig 3B, no axis labels.

Fig 5A,B: conditions “A,B,C” not clearly labeled so difficult to interpret.

References:

Some references are duplicated (#13 and 43).

Methods section:

Should be reorganized to follow presented results. Organize to ZIKV experiments, then HIV, then SARS2 experiments.
