# Supplementary material for: B Cell Receptor’s function in virus entry: Anti-SARS-CoV-2 B cell receptors can mediate viral entry in an ACE2-independent mechanism
Source: PLoS Pathog. 2026 Feb 6;22(2):e1013946. doi: 10.1371/journal.ppat.1013946 (PMC12893656; doi:10.1371/journal.ppat.1013946)
Supplement: Attachment — Submitted filename: 122925 Responses to reviewers.docx [file ppat.1013946.s006.docx]

**Responses to reviewers' comments**
Italic font indicates the comments of the reviewers, and blue regular font for author responses.

**RESPONSE TO SPECIFIC COMMENTS**

**Journal Requirements**

*1) Please ensure that the CRediT author contributions listed for every co-author are completed accurately and in full.
At this stage, the following Authors/Authors require contributions: Rene Larios, Md Belal Hossain, Rebecca Brown, Arjit Vijey Jeyachandran, Angel Elma Abu, Masakazu Kamata, Steve Cole, Ting-Ting Wu, Kenneth Dorshkind, Vaithilingaraja Arumugaswami, and Kouki Morizono. Please ensure that the full contributions of each author are acknowledged in the "Add/Edit/Remove Authors" section of our submission form.
The list of CRediT author contributions may be found here:* [*https://journals.plos.org/plospathogens/s/authorship#loc-author-contributions*](https://journals.plos.org/plospathogens/s/authorship#loc-author-contributions)
As requested, we have updated the CRediT author contributions for all listed co-authors in the submission form. We have ensured that the roles assigned to each individual accurately and comprehensively reflect their specific contributions to the study, in accordance with the PLOS Pathogens guidelines.

*2) Please upload all main figures as separate Figure files in .tif or .eps format. For more information about how to convert and format your figure files please see our guidelines:* 
[*https://journals.plos.org/plospathogens/s/figures*](https://journals.plos.org/plospathogens/s/figures)
In accordance with the journal’s formatting guidelines, we have converted all main figures to the required **.tif/.eps format**. These have been uploaded as separate files via the submission portal. We have also ensured that all files meet the specified resolution and compression requirements to maintain high-quality reproduction.

*3) We notice that your supplementary Figures are included in the manuscript file. Please remove them and upload them with the file type 'Supporting Information'. Please ensure that each Supporting Information file has a legend listed in the manuscript after the references list.*
As requested, the supplementary figures have been separated from the main manuscript and re-uploaded as 'Supporting Information' files. The corresponding legends have been moved to the end of the manuscript, following the reference list, to ensure compliance with the journal's formatting requirements.

*4) Some material included in your submission may be copyrighted. According to PLOSu2019s copyright policy, authors who use figures or other material (e.g., graphics, clipart, maps) from another author or copyright holder must demonstrate or obtain permission to publish this material under the Creative Commons Attribution 4.0 International (CC BY 4.0) License used by PLOS journals. Please closely review the details of PLOSu2019s copyright requirements here: PLOS Licenses and Copyright. If you need to request permissions from a copyright holder, you may use PLOS's Copyright Content Permission form.
Please respond directly to this email and provide any known details concerning your material's license terms and permissions required for reuse, even if you have not yet obtained copyright permissions or are unsure of your material's copyright compatibility. Once you have responded and addressed all other outstanding technical requirements, you may resubmit your manuscript within Editorial Manager.*

In response to the query regarding copyrighted material, we confirm that all images in Figure 6 were produced entirely by the authors. As these are original graphics, they do not require third-party copyright permissions and are submitted under the CC BY 4.0 license.

*5) In the online submission form, you indicated that All data in this manuscript, along with detailed experimental records, are available upon request to Kouki Morizono.. All PLOS journals now require all data underlying the findings described in their manuscript to be freely available to other researchers, either
1. In a public repository
2. Within the manuscript itself
3. Uploaded as supplementary information.
This policy applies to all data except where public deposition would breach compliance with the protocol approved by your research ethics board. If your data cannot be made publicly available for ethical or legal reasons (e.g., public availability would compromise patient privacy), please explain your reasons by return email and your exemption request will be escalated to the editor for approval. Your exemption request will be handled independently and will not hold up the peer review process, but will need to be resolved should your manuscript be accepted for publication. One of the Editorial team will then be in touch if there are any issues.*
The previous statement regarding 'data available upon request' was originally intended to express our willingness to share raw laboratory records; however, to meet the journal's requirements for immediate accessibility, we have removed that language. We have replaced it with the following statement on line 605: 'All relevant data are within the manuscript and its Supporting Information files.'

*6) Please amend your detailed Financial Disclosure statement. This is published with the article. It must therefore be completed in full sentences and contain the exact wording you wish to be published.*

*1) State the initials, alongside each funding source, of each author to receive each grant. For example: "This work was supported by* *the National Institutes of Health (####### to AM; ###### to CJ) and the National Science Foundation (###### to AM)."*

*2) State what role the funders took in the study. If the funders had no role in your study, please state: "**The funders had no role in study design, data collection and analysis, decision to publish, or preparation of the manuscript."*

*3) If any authors received a salary from any of your funders, please state which authors and which funders.*

*If you did not receive any funding for this study, please simply state: u201cThe authors received no specific funding for this work. u201d*

We have amended the Financial Disclosure statement as requested. We have ensured the statement consists of full sentences, includes author initials for each grant, and clarifies that the funders had no role in the study or manuscript preparation. The revised statement is provided below and has been updated in the manuscript submission system.

Line 613-620

This work was supported by the National Institutes of Health (R21AI095004, R01AI108400, R01AI145044, and U19AI149504 to K.M.; R01EY032149, U19AI125357, R01AI163216, and R01DK132735 to V.A.) and the California Institute for Regenerative Medicine (DISC2-16725 to K.M.). Additional support was provided by the UCLA AIDS Institute, the Center for AIDS Research- University of California Los Angeles, the James B. Pendleton Charitable Trust, and the McCarthy Family Foundation. The funders had no role in study design, data collection and analysis, decision to publish, or preparation of the manuscript.

**EDITOR**

*However, they noted that key technical details and rigor were lacking in several portions of the manuscript as currently presented.*
We have addressed this concern by providing extensive detail in the Materials and Methods section, expanding the Figure Legends for clarity, and refining technical points within the main text. Our point-by-point response to the reviewers (provided below) further details how we have satisfied these specific editorial requests. We believe these revisions significantly enhance the transparency and rigor of our methodology.

*Additionally, the authors need to carefully consider the accuracy of several statements regarding the infectibility of B cells by SARS-CoV-2, as the studies currently cited do not directly support these claims.*
We have revised the relevant sections of the manuscript to ensure that our claims regarding SARS-CoV-2 and B cells are supported by direct experimental evidence. All overstatements have been corrected to reflect a more nuanced and accurate interpretation of the cited research as detailed in our point-by-point response below.

**REVIEWER 1**

*In several locations, data from two independent experiments are shown, rather than collation of the data. Why is this? It appears that those studies where this is shown were done twice. The standard in the field is performing experiments three independent times and then pooling the data with standard deviation or standard error bars to provide the reader with an estimate of the variation.*

We thank the reviewer for this comment and agree that pooling data across independent experiments with appropriate estimates of variability is the preferred standard when feasible. The figures highlighted by the reviewer represent two distinct experimental contexts with different sources of biological variability: primary human donor samples (Fig. 1E) and in vivo animal studies (Fig. 2B).

Fig 1E (primary human cells)

For Fig. 1E, experiments were performed using primary human cells derived from independent donors, representing biological replicates rather than technical repeats. Given the well-established donor-to-donor heterogeneity of primary human immune cells, we initially displayed donor-stratified data to transparently illustrate biological variability while demonstrating consistency in the direction of effect.

In response to the reviewer’s comment, we performed a linear mixed-effects analysis with condition as a fixed effect and donor as a random effect. No significant difference was observed between None and Control (p = 0.31), whereas both ZKA190 (p = 0.0011) and ZKA230 (p = 0.0028) showed highly significant increases relative to None. These results confirm that the observed effects are robust and remain statistically significant after accounting for inter-donor variability.

To further demonstrate reproducibility, we additionally performed within-donor pairwise analyses using Welch’s two-sample t-tests with pre-specified contrasts versus None:

Donor 1:

None vs Control p=0.57

ZKA190 significantly increased infection relative to None, p = 0.034.

ZKA230 significantly increased infection relative to None, p = 0.05.

Donor 2:

None vs Control, p=0.45

ZKA190 also significantly increased infection relative to none, p = 0.004).

ZKA230 significantly increased infection relative to None, p = 0.005).

Pooled analysis (Experiments 1 + 2): When data were combined across both experiments, ZKA190 remained highly significantly different from none, p = 5.4 × 10⁻⁴.

Pooled None v Control was not significant, p=0.32

These statistical analyses are now included in the figure legends.

Figure 2B (in vivo animal studies )

Figure 2B represents two independent in vivo animal experiments, each performed separately. These experiments were initially shown separately to demonstrate reproducibility across independent animal cohorts, given the known biological variability inherent to in vivo infection models.

In response to the reviewer’s request, we have now performed formal statistical analyses both within each experiment and on the pooled dataset:

Experiment 1: ZKA190 significantly increased infection relative to control

(Welch’s two-sample t-test, p = 0.0022).

Experiment 2: ZKA190 also significantly increased infection relative to control

(Welch’s two-sample t-test, p = 0.0127).

Pooled analysis (Experiments 1 + 2): When data were combined across both experiments, ZKA190 remained highly significantly different from control

(Welch’s two-sample t-test, p = 2.9 × 10⁻⁴).

As a robustness check, we additionally analyzed the pooled in vivo data using a mixed-effects model with experiment treated as a random effect, which yielded the same conclusion, with ZKA190 remaining a highly significant predictor of increased infection (p < 0.01).

These statistical analyses are now included in the figure legends.

Line 681-688
We performed within-donor pairwise analyses using Welch’s two-sample t-tests with pre-specified contrasts versus None: Donor 1: None vs Control p=0.57, ZKA190 significantly increased infection relative to None, p = 0.034. ZKA230 significantly increased infection relative to None, p = 0.05. Donor 2: None vs Control, p=0.45. ZKA190 also significantly increased infection relative to none, p = 0.004. ZKA230 significantly increased infection relative to None, p = 0.005. Pooled analysis (Experiments 1 + 2): When data were combined across both experiments, ZKA190 and ZKA230 remained highly significantly different from none, p < 1 × 10⁻^3^. Pooled None vs Control was not significant, p=0.32

Line 699-706
Statistical analyses were performed both within each experiment and on the pooled dataset: Experiment 1: ZKA190 significantly increased infection relative to control (Welch’s two-sample t-test, p = 0.0022). Experiment 2: ZKA190 also significantly increased infection relative to control. (Welch’s two-sample t-test, p = 0.0127). Pooled analysis (Experiments 1 + 2): When data were combined across both experiments, ZKA190 remained highly significantly different from control (Welch’s two-sample t-test, p < 1 × 10⁻3). We additionally analyzed the pooled in vivo data using a mixed-effects model with experiment treated as a random effect, and ZKA190 remaining a highly significant predictor of increased infection (p < 0.01).

*The Delorey paper (reference #14) is misrepresented here as providing supporting evidence that “SARS-CoV-2 are known to infect B cells in patients” (line 90). That Nature paper investigating SARS-CoV-2 RNA in the lung of patients succumbing to COVID does not state that active viral replication is occurring in B cells in their patient samples. Instead, SARS-CoV-2 RNA in B cells of lung from acutely infected patients was infrequent and the authors suggested “immune cell engulfment and virions or virally infected cells attached to the cell surface” were likely responsible for their observation that ACE2-null cells had associated virus.*

We thank the reviewer for this critical clarification regarding the interpretation of Reference (Delorey et al., Nature, 2021). The reviewer is correct that the original text on line 90, "SARS-CoV-2 are known to infect B cells in patients," misrepresents the findings of the Delorey paper. The authors of that study, while detecting SARS-CoV-2 RNA associated with B cells in the lung, did not conclude that these cells were productively infected or supporting active viral replication. Instead, they proposed alternative mechanisms, such as engulfment of virions or infected cells, to explain the presence of viral RNA in these ACE2-negative cells. We agree that it is essential to accurately reflect the nuanced conclusions of published literature. We have revised the manuscript to accurately align with the findings of the Delorey et al. study.

Line 91-97

In addition to flaviviruses, other types of enveloped viruses, such as SARS-CoV-2, have been reported to infect B cells, in patients at least in vitro [13]. In acutely infected COVID-19 patients, SARS-CoV-2 RNA has also been detected in association with B cells in the lungs [14]; However, this viral signal was infrequent, and the authors suggested that it likely reflects immune cell engulfment or surface-associated virions rather than productive infection. Thus, their molecular mechanisms have not yet been elucidated.

*The statement made starting on line 94 implies that EBOV glycoprotein does not require binding of endosome NPC1 for subsequent fusion events. This is a misstatement and should be corrected.*

We appreciate the reviewer’s correction. We apologize for the misstatement regarding the EBOV glycoprotein’s requirement for NPC1. We have amended the text by removing EBOV from the sentence to maintain technical accuracy.

*Virus stocks used in these studies were not purified. Instead, the authors concentrated stock with a 100 kDa cut off concentrator. This approach may be concentrating proteins present in the supernatant that are >50 kDa that facilitate virion uptake. Virions that have been separated from host and media proteins should be tested in the binding and infection assays.*

Thank you for raising the important concern regarding the potential co-concentration of host/media proteins that could influence virion uptake, particularly for the experiments using the low-titer ZIKV replicon system. We agree that separating the virions from host and media proteins is critical to confidently attribute the observed effects to the virus and our binding/infection factors. For the replication-competent virus experiments, we did not concentrate the stocks, as their high titers allowed us to perform the assays directly, minimizing the issue of co-concentrated proteins. For the experiments involving the ZIKV replicon-based virus (which necessitated concentration due to lower initial titers, using a 100 kDa cut-off concentrator), we performed additional purification steps to address the reviewer's concern.

Initial Attempt (Ultracentrifugation): We attempted purification via ultracentrifugation, but consistent with previously reported findings, this method significantly eliminated the virus titer, rendering the stock unusable for the functional assays.

Successful Purification (Size Exclusion Chromatography): We subsequently purified the concentrated virus stock using Size Exclusion Chromatography (SEC) with a Sephacryl S-500 column to eliminate host proteins and complexes smaller than 500 kDa, thereby separating the virions from most host and media components, including those. We then re-tested the binding using this purified, fluorescently-labeled ZIKV replicon-based virus. As shown in Figure 1A, the results confirmed that the anti-ZIKV BCRs mediate the binding of this purified virus stock, demonstrating that the observed attachment effect is intrinsic to the virion interaction and not due to co-concentrated proteins. We believe these results effectively confirm that anti-ZIKV BCRs facilitate ZIKV infection primarily by mediating the attachment of the virion itself.

Accordingly, we have updated the Materials and Methods section regarding virus purification as below.

Line 482-486

Unincorporated Vybrant DiD was then removed by four serial passes through size exclusion chromatography using 40-kD cut-off Zeba columns (ThermoFisher) and by adsorption on Ramos cells, followed by size exclusion chromatography with a Sephacryl S-500 column (Cytiva, Marlborough, MA) to eliminate host proteins and complexes smaller than 500 kDa.

*To truly demonstrate productive virus infection (and exclude simple cellular internalization of virions), virus titration should be performed in supernatants taken from the infected cells.*
We appreciate the reviewer's comment regarding the necessity of demonstrating productive virus infection and excluding simple virion internalization. To definitively confirm this, we performed virus titration on the supernatants of the infected cells, which is the established method to quantify infectious progeny virus release. We infected the antiviral BCR-expressing cells (as well as control cells) with Zika Virus (ZIKV) or SARS-CoV-2. The cell supernatants were collected two days post-infection and subsequently titrated on highly susceptible Vero E6 cells. These new titration results, now presented in Figure 1D (for ZIKV) and Figure 5D (for SARS-CoV-2), confirmed the release of infectious viral particles from the antiviral BCR-expressing cells. This data, therefore, establishes that the cells expressing antiviral BCR are capable of supporting a productive viral infection.

Line 152-157

To confirm productive ZIKV infection, supernatants from the cells described in Figs 1B and C were used to inoculate VeroE6 indicator cells. ZIKV infection was subsequently detected in VeroE6 cells via intracellular staining of the ZIKV E protein (Fig. 1D), confirming the presence of infectious virus in the supernatants of Ramos and Sp2/0 cells expressing anti-ZIKV BCRs.

Line 300-305

To confirm productive SARS-CoV-2 infection, supernatants from the anti-SARS-CoV-2 BCR-expressing cells (Fig. 5C) were used to inoculate VeroE6 indicator cells. Infection was subsequently confirmed in the VeroE6 cells by the detection of SARS-CoV-2 N1 and N2 genes (Fig. 5D), verifying the presence of infectious virus in the original supernatants.

*The discussion implies that non-neutralizing Abs would like not be able to mediate entry of ZIKV or SARS-CoV-2 since those Abs would not bind to the receptor binding domain and potentially conformationally change the viral glycoprotein. However, this could be directly tested by the authors. Testing of non-neutralizing Ab would be insightful. Are the Abs that mediate fusion events known to alter conformation of the respective viral glycoproteins?*

We appreciate the reviewer's insightful question regarding the role of non-neutralizing antibodies and glycoprotein conformational change in the mechanism of antiviral BCR-mediated virus entry.

ZIKV Entry Mechanism: Non-neutralizing antibodies against ZIKV have previously been shown to mediate ADE because exposure to low pH can induce the necessary conformational changes in ZIKV for fusion. We concur that receptor-binding domain (RBD) binding is not a prerequisite for ZIKV entry in the context of BCR-mediated infection. ZIKV can also utilize DC-SIGN, which mediates infection by binding to the N-glycans present on the ZIKV E protein, independent of the RBD itself. As previously reported by our group and others, this DC-SIGN-mediated mechanism highlights that binding outside the primary neutralization epitope is sufficient for viral internalization and infection. We have clarified this mechanistic rationale in the Discussion section of the revised manuscript as follows:

Line 325-332

We utilized BCRs derived from ZIKV-neutralizing antibodies. Since DC-SIGN, which binds the N-glycans of the ZIKV E protein, can facilitate viral attachment and infection, neutralizing activity may not be strictly required for BCR-mediated entry, as observed for ADE mediated by non-neutralizing antibodies against ZIKV. However, the discrepancy in the ability of two specific anti-ZIKV BCRs, ZKA190 and ZKA230, to mediate virus binding and infection in Ramos cells (Figure 1A and B) suggests that additional factors—such as the capacity of a BCR to prevent pH-dependent conformational changes in the E protein—influence the efficiency of BCR-mediated viral entry.

Testing Non-Neutralizing Antibodies: We acknowledge that the investigation of BCRs derived from a panel of antibodies with various neutralizing potencies, particularly non-neutralizing antibodies, would be highly informative and provide a deeper understanding of the entry mechanism. However, the primary objective of the current study is to establish the novel finding that antiviral BCRs can be utilized as a route for the entry of ZIKV and SARS-CoV-2. Further detailed mechanistic investigation involving a comprehensive panel of BCRs derived from non-neutralizing antibodies would constitute a significant expansion of the study's scope, requiring extensive new data generation. Given the current focus, we respectfully defer this comprehensive mechanistic experiment for a future dedicated study.

*More details about the anti-Zika monoclonals (mAbs) are needed. The authors indicate that these mAbs are neutralizing in solution but mediate entry into B cell lines when present on the plasma membrane. Do these soluble antibodies when bound by Fc or other cell surface attachment mechanism mediate virus uptake? Do non-neutralizing mAbs also internalize virus?*

We have detailed the characteristics of these antibodies within the manuscript. Specifically, we noted the binding domains of these mAbs and clarified that they bind to Fc Receptors and mediate ADE, based on studies by other groups. Furthermore, non-neutralizing mAbs are also reported to mediate ADE, a crucial point which we have explicitly discussed in the Discussion section as we mentioned above. The added sentences are below.

Line 141-145

We engineered anti-ZIKV BCRs derived from the antibodies ZKA190 and ZKA230. While ZKA190 targets domain III of the E protein, ZKA230 recognizes quaternary epitopes present on infectious virions but absent on soluble proteins. Although both antibodies potently neutralize ZIKV in Fc receptor-negative cells, they are known to induce antibody-dependent enhancement (ADE) in cells expressing Fc receptors.

*The authors conclude from Panels B and C as well as panel E that ZIKV infection is occurring, but this is not convincing. More extensive evidence that this is a true infection is needed. To truly demonstrate productive virus infection (and exclude simple virus attachment to cells or cellular internalization of virions without subsequent productive events), virus titration should be performed in supernatants taken from the infected cells. Alternatively (or in addition), the authors should demonstrate that replication of their infectious virus is occurring over a time course of infection with increasing numbers of infected cells over time.*

We agree that definitive evidence demonstrating infectious virus production is crucial to support our conclusions. To address this, we have conducted the critical experiment suggested by the reviewer: we performed a virus infection using the supernatants collected from the BCR expressing cells following ZIKV infection. The results of this infection, which confirm the release of infectious progeny virions, are now presented in the revised manuscript as Figure 1D as described above. This new data decisively demonstrates productive virus infection and excludes the possibility of mere virus attachment or non-productive internalization.

*The figure legend states that flow shown in panel D shows primary human B cells, but the text starting on line 142 indicates that the study shown in panel D is in a B cell line. Please correct which ever statement is incorrect.*

We thank the reviewer for identifying this critical inconsistency. The statement has been corrected in both the figure legend and the corresponding text to accurately reflect the cell type used in the study presented in Panel D.

*Fig. 2
In the flow panel shown in A, the B cells expressing high levels of hIgG1 appears to be several fold greater in the ectopic BCR panel. Could the increase in viral antigen positive cells be due to a greater number of B cells expressing high hIgG1 in that treatment group rather than due to expression of the ZIKV specific Ab?
Intravenous infection with several different concentration of ZIKV would make the data more*

To address this potential confounder, we have incorporated quantitative data showing the average and standard deviation (SD) of hIgG1 expressing cells in Figure 2A. Following re-collation of all data, we confirm that the hIgG1 expression levels between the two study groups are not differentially expressed to an extent that would account for the difference in ZIKV antigen positivity, thereby confirming that the increase is attributable to the ZIKV-specific antibody expression.

*Fig. 3.
The authors speculate on why eCD4-Ig BCR binding to HIV does not mediate the required conformational changes in Env for CXCR4 binding and subsequent fusion events. Such proposed studies could be performed.*
We appreciate the reviewer's insightful suggestion to explore the mechanistic reasons why eCD4-Ig BCR binding to HIV Env fails to mediate the necessary conformational changes for co-receptor binding and subsequent fusion. We agree that shortening the BCR stalk domain or creating a monomeric BCR are conceptually viable approaches to test our proposed hypotheses. However, these experimental manipulations pose significant technical challenges, as such structural modifications would likely disrupt the interaction with essential chaperone molecules CD79α and β, resulting in severely compromised or absent cell surface expression of the BCR. Furthermore, we considered that based on previous findings showing that CD4 and CXCR4/CCR5 associate on the membrane to facilitate HIV infection, a lack of such functional association between the BCR and the co-receptors could also contribute to the failure of eCD4-Ig BCR to mediate HIV-1 infection. Given the significant technical complexities, the need for extensive controls, and the inherent difficulty in definitively comprehending the possibilities of the underlying mechanistic failure in the context of the current study, we have concluded that investigating these specific molecular reasons is beyond the current scope and focus of this manuscript. We have revised the relevant section in the Discussion to clearly state the limitations and frame our hypotheses as avenues for future investigation. As below.

Line 372-378

3: While the association of CD4 with CCR5 or CXCR4 prior to viral attachment is known to enhance HIV-1 infection [71], the eCD4 BCR might be unable to associate with these co-receptors. This inability may persist both before and after HIV-1 binding.

Essential next steps would include optimizing eCD4 BCR stalk length for closer membrane proximity, while maintaining CD79 interaction, and exploring monomeric variants and/or co-receptor association to identify the specific mechanistic barriers to BCR-mediated HIV-1 entry.

*Fig. 5.
The ABC treatments shown in panels A and B are not well described. Please elaborate more clearly in the figure legend and main text if you wish to use this terminology.
Similar to the previous comment about the ZIKV studies, demonstration of production of infectious virus from CB6 BCR and/or REGN BCR expressing cells would enhance findings, making it more convincing that these cell surface bound antibodies are sufficient to mediate productive infection.*

We acknowledge the lack of clarity regarding the ABC treatments in Figure 5, Panels A and B. We have revised both the figure legend and the main text to elaborate in detail on the components and procedures involved in the ABC treatments, ensuring the terminology is thoroughly defined for the reader.

Line 760-772

A) 293T cells, with or without ectopic expression of ACE2, were infected with SARS-CoV-2 VLPs carrying a firefly luciferase reporter gene. These infections were performed in the presence or absence of control antibodies (Block A), anti-ACE2 antibodies (Block B), or the protease inhibitor E64d (Block C). Two days post-infection, viral infection was quantified by measuring firefly luciferase activity in the lysates of the infected cells. N=3 and averages are shown with SD (error bar). This experiment was repeated twice in singlicate and once in triplicate, and the results of the triplicated experiments are shown. B) Infection of CD79 293T cells, with or without ectopic expression of anti-SARS-CoV-2 BCR (REGN10933 and CB6), with SARS-CoV-2 VLP carrying a firefly luciferase reporter gene in the presence and absence of control (Block A) and anti-ACE2 antibodies (Block B), or E64d (Block C). N=3 and averages are shown with SD (error bar). Two days post-infection, viral infection was quantified by measuring firefly luciferase activity in the lysates of the infected cells. This experiment was repeated twice in singlicate and once in triplicate, and the results of the triplicated experiments are shown.

To address the need for definitive evidence of productive infection, we conducted the suggested titration experiments with SARS-CoV-2. We infected the antiviral BCR-expressing cells (and controls), collected the supernatants two days post-infection, and titrated them on Vero E6 cells. These new titration results, now presented in Figure 5D, confirmed the release of infectious viral particles from the antiviral BCR-expressing cells, thereby establishing that the cells expressing antiviral BCR are fully capable of supporting a productive viral infection.

*Major Issues: Key Experiments Required for Acceptance*

*Reviewer #1: The authors need to demonstrate more clearly that the membrane bound IgMs are mediating productive virus infection. Additional more mechanistic studies would benefit the manuscript.*

We thank the reviewer for this constructive feedback. We agree that demonstrating productive infection is crucial to supporting our claims.

To address this, we performed additional experiments specifically focused on ZIKV and SARS-CoV-2. Our new data, now included in Figs 1D (ZIKV) and Figure 5D (SARS-CoV-2), clearly demonstrate that antiviral BCRs indeed mediate productive viral infection for these pathogens.

Regarding the suggestion for further mechanistic studies involving anti-HIV-1 BCR derivatives: while we acknowledge the value of such an investigation, we consider it to be beyond the primary scope of this manuscript. Engineering BCR variants capable of mediating HIV-1 entry would require a significant investment of time and resources that exceed the current focus of our work as detailed in our previous responses and the revised Discussion. We believe the data provided for ZIKV and SARS-CoV-2 sufficiently establish the phenomenon of BCR-mediated infection as the central focus of this paper.

**REVIEWER 2**

*Across the different experimental systems there are several variables for each and this lends itself to overreliance on blanket terms like “infection” , “attachment”, “fusion”. These terms are not clearly enough defined in the legends or on the graphs in figures, prompting a deep dive in the methods to understand the experimental setup. For example, how is infection defined in Fig 1B,C? This is unclear in results text or legend. The methods (lines 469-470) mention staining for flavivirus group antigen (is this 4G2?) in permeabilized cells, so this could just be viral uptake rather than infection.*

We have clarified in the legend that "infection", “attachment”, and “fusion” refers to the detection of de novo viral protein synthesis. As the reviewer correctly surmised, we used the 4G2 antibody (anti-flavivirus envelope) for intracellular staining of ZIKV infected cells. To distinguish bona fide infection from simple viral uptake, we have included a representative flow cytometric profile in Fig S1A and B, S2A and B, and S3C and D. The strong staining of the ZIKV E protein in a distinct subpopulation indicates the production of new viral proteins rather than the presence of incoming virus, which typically results in a dim, uniform staining of the entire population. To definitely distinguish bona fide infection from simple viral uptake, we have added Figure 1D, which shows the production of progeny viruses in the supernatant over time. This evidence of viral replication confirms that the 4G2-positive signal represents a productive infection rather than sequestered inoculum.

We also updated the figure legends for readers to easily understand the assays used to evaluate “attachment” “fusion” “infection” and “transduction”.

Line 658-662

Cells were infected with replication-competent ZIKV at a multiplicity of infection (MOI) of 10. The viral titer was determined by TCID50 assay using VeroE6 cells. At 48 hours post-infection, ZIKV infection was quantified via flow cytometry following intracellular staining of the ZIKV E protein using an Alexa 647-conjugated anti-ZIKV E protein antibody (4G2).

Line 676-680

The cells were infected with a ZIKV replicon expressing EGFP at a multiplicity of infection (MOI) of 10 with the replicon titer determined by flow cytometry using VeroE6 cells. At 48 hours post-infection, ZIKV infection, defined by EGFP expression, was quantified via flow cytometry in untransduced CD19-positive B cells (None) and B cells expressing either the control or anti-ZIKV BCR (Flag tag-positive).

Line 690-697

Four-to-six-week-old IFNAR KO male or female mice received retro orbital injection of 2.2 1LFlag1L (a modified Sindbis virus envelope designed for B-cell targeting in vivo) pseudotype [4], expressing ZIKV190 BCR or control BCR (anti-HIV-1 3BNC117). Four days post-lentiviral injection, mice were retro-orbitally injected with 150 µL of ZIKV replicon (3X106 infectious units). Sixteen-hours post-injection, spleen cells were isolated and stained with anti-CD19 PE, anti-CD3 BV421, and Alexa 647 anti-hIgG Fc. The percentages of EGFP-positive cells within the CD19+ hIgG+ population were then analyzed by flow cytometry.

Line 712-715

CD79 293T and CD79 CXCR4 293T cells, with or without transfection of an expression vector for human CD4 were incubated with Scarlet-labeled HIV-1 gp160 pseudotyped lentiviral vectors for one hour at 37°C. Virus binding was analyzed by flow cytometry.

Line 716-718

Two days after infection, infected cells were identified via intracellular staining using a PE-conjugated antibody against the HIV-1 Gag protein, followed by flow cytometric analysis.

Line 722-724

The cells were incubated with Scarlet-labeled HIV-1 gp160 pseudotyped lentiviral vectors for one hour at 37°C. Virus binding was analyzed by flow cytometry.

Line 726-728

Two days after infection, infected cells were identified via intracellular staining using a PE-conjugated antibody against the HIV-1 Gag protein, followed by flow cytometric analysis.

Line 738-740

The cells were incubated with the vector for 1 hour at 37°C, and Scarlet-positive populations were quantified as a measure of virus binding via flow cytometry.

Line 742-745

The cells and virus were incubated for 1 hour at 37°C. The cells were then incubated with CCF4-AM for 1 hour at RT. Using flow cytometry, entry of virus is quantitated by cleavage of CCF4-AM in the cytoplasm of target cells. The positive signal in the BV421 channel was measured as virus fusion.

Line 747-751

Transduction of 293T cells with or without ectopic expression of TIM-1, ACE2, as well as CD79 293T cells with ectopic expression of control, or anti-SARS-CoV-2 BCR with lentiviral vector pseudotyped with S protein, carrying an EGFP transgene. Three days after transduction, transduction was quantified by analyzing EGFP transgene expression using flow cytometry.

Line 752-756

Transduction of CD79 Sp2/0 cells with or without ectopic expression of ACE2, control or anti-SARS-CoV-2 BCR with lentiviral vector pseudotyped with S protein carrying an EGFP transgene in the absence and or presence of Nevirapine (10 µM). Three days after transduction, transduction was quantified by analyzing EGFP transgene expression using flow cytometry.

Line 760-771

293T cells, with or without ectopic expression of ACE2, were infected with SARS-CoV-2 VLPs carrying a firefly luciferase reporter gene. These infections were performed in the presence or absence of control antibodies (Block A), anti-ACE2 antibodies (Block B), or the protease inhibitor E64d (Block C). Two days post-infection, viral infection was quantified by measuring firefly luciferase activity in the lysates of the infected cells. N=3 and averages are shown with SD (error bar). This experiment was repeated twice in singlicate and once in triplicate, and the results of the triplicated experiments are shown. B) Infection of CD79 293T cells, with or without ectopic expression of anti-SARS-CoV-2 BCR (REGN10933 and CB6), with SARS-CoV-2 VLP carrying a firefly luciferase reporter gene in the presence and absence of control (Block A) and anti-ACE2 antibodies (Block B), or E64d (Block C). N=3 and averages are shown with SD (error bar). Two days post-infection, viral infection was quantified by measuring firefly luciferase activity in the lysates of the infected cells.

Line 773-777

CD79 Sp2/0 cells, with or without ectopic expression of ACE2, a control BCR, or an anti-SARS-CoV-2 BCR (ScFv form), were infected with either live or heat-inactivated SARS-CoV-2. Two days after infection, the RNA was isolated from infected cells and the copy numbers of mouse GAPDH and SARS-CoV-2 N1 and N2 RNA copy numbers were quantified by digital droplet PCR.

*Figure 2A: Looking at very small effects. Even in IFNARKO mice, there is limited transduction of BCRs (percentages should be given) and of those transduced cells, only a small fraction of these become GFP+ (3-6%) following in vivo ZIKV replicon administration via i.v. injection. Though a t-test indicates statistical significance, the physiological relevance is unclear.*

We thank the reviewer for this observation and have now included the specific percentages of BCR-expressing cells in Figure 2A. While we previously observed approximately 2% transduction efficiency in splenic B cells using EGFP-expressing lentiviral vectors, we detected only 0.3-0.6% of cells expressing the lentiviral BCR. This difference is likely due to the fact that lentiviral vectors expressing the BCR transgene (2300 bp) yield lower titers compared to those carrying smaller transgenes like EGFP (780 bp). Additionally, detecting BCR expression via antibody staining is inherently less sensitive than detecting direct fluorescent protein expression. Although the percentage of BCR-expressing cells is low, we acquired data from 1X10^7^ total splenocytes via flow cytometry to ensure a sufficient number of events for a robust statistical analysis of infection percentages.

The relatively low frequency of GFP+ cells (3–6%) is primarily due to the inherent constraints of our in vivo model, specifically that the ZIKV replicon undergoes only a single cycle of infection and cannot spread to neighboring cells. Furthermore, the intravenous administration of the replicon leads to systemic distribution and the potential sequestering of viral particles in other organs, all while facing an overwhelming population of approximately 100 million total splenocytes per mouse.

To overcome these biological hurdles and ensure our data remained within a statistically reliable range, we utilized concentrated ZIKV replicon stocks. While we acknowledge that the absolute percentages are modest, the consistent statistical significance achieved across experimental replicates demonstrates a genuine biological effect. Under currently available experimental settings, it is technically difficult to further increase these infection percentages without compromising the physiological state of the model.

*In Figures 4 and 5 the message is that SARS2-specific BCRs facilitate binding and fusion of SARS2 pseudotyped lentivirus and infection with SARS2 in 293T cells to the same extent as ACE2. Furthermore, cathepsin L activity is similarly required for ACE2 or BCR-mediated infection. The labels A,B,C in Fig 5A,B are not clearly indicated so difficult to confirm what is being tested.*

We have revised the figure legend to elaborate in detail on the components and procedures involved in the ABC treatments, ensuring the terminology is thoroughly defined for the reader.

Line 761-769

These infections were performed in the presence or absence of control antibodies (Block A), anti-ACE2 antibodies (Block B), or the protease inhibitor E64d (Block C). Two days post-infection, viral infection was quantified by measuring firefly luciferase activity in the lysates of the infected cells. N=3 and averages are shown with SD (error bar). This experiment was repeated twice in singlicate and once in triplicate, and the results of the triplicated experiments are shown. B) Infection of CD79 293T cells, with or without ectopic expression of anti-SARS-CoV-2 BCR (REGN10933 and CB6), with SARS-CoV-2 VLP carrying a firefly luciferase reporter gene in the presence and absence of control (Block A) and anti-ACE2 antibodies (Block B), or E64d (Block C).

*On line 206 the authors cite refs 14, and 43 (which is a duplicate of ref 13) as evidence that SARS-CoV-2 infects B cells in vivo. Ref 14 is an atlas of SARS2 RNA-containing lung cells during COVID-19 wherein phagocytic cells in general have SARS2 RNA, with B/plasma cells having a weak representation. Ref 13/43 relies mainly on dsRNA staining (specific for SARS2?, not described) and SARS2 antigen detected with serum from COVID-19 patient. So these references are not convincing for the rationale.*

We apologize for this oversight of the reference duplication. We have carefully re-examined the cited study and agree with the reviewer that it does not provide a definitive conclusion regarding SARS-CoV-2 infection in B cells. We removed reference 13/43 to ensure our claims accurately reflect the current literature. We have revised the corresponding statements in Revised manuscript to reflect the exact findings of the reference.

Line 91-97

In addition to flaviviruses, other types of enveloped viruses, such as SARS-CoV-2, have been reported to infect B cells, in patients at least in vitro [13]. In acutely infected COVID-19 patients, SARS-CoV-2 RNA has also been detected in association with B cells in the lungs [14]; However, this viral signal was infrequent, and the authors suggested that it likely reflects immune cell engulfment or surface-associated virions rather than productive infection. Thus, their molecular mechanisms have not yet been elucidated.

*Furthermore, SARS2-specific BCR-bearing memory B cells which expand following exposure or vaccination could potentially mediate increasing viral load and disease following SARS2 challenge, but this is not borne out, like what happens for dengue.*

We thank the reviewer for this insightful observation. While the theoretical potential for memory B cell expansion to facilitate viral replication exists, we agree that this phenomenon has not been observed for SARS-CoV-2. We have incorporated this point into our revised Discussion as below.

Line 379-382

Although SARS-CoV-2-specific BCR-bearing memory B cells, which expand following exposure or vaccination, could potentially facilitate increased viral replication, particularly within the B-cell population, no previous studies have reported the existence of such a phenomenon. Thus, it remains uncertain whether the induction of antiviral BCRs on B cells through vaccination or infection results in enhanced viral entry and replication.

*Other issues:*

*Line 117: dengue virus doesn’t “induce conformational changes”, rather the lowering of pH upon endocytosis does.*

We have revised the sentence in the manuscript to reflect that conformational changes in the Dengue virus envelope proteins and subsequent membrane fusion are triggered by the acidic environment of the endosome. The updated are as below.

Line 123-125

Conformational changes of Dengue virus envelope proteins and subsequent fusion are triggered by the low pH environment in the endosome.

*Line 120: cannot say “universally” based on one other manuscript.*

We have removed the word "universally" from the sentence in the revised manuscript to avoid overgeneralization based on the cited literature.

*Line 126: what is meant by “facilitate”? rather in the Influenza paper, BCR signaling sensitizes cell for infection and for RSV, the infection is of a rather narrow subset of B cell and is through CX3CR1.*

We have revised the main text to more accurately define how antiviral BCRs "facilitate" infection, specifically addressing the mechanisms described in the cited studies.

Line 133-138

Although antiviral BCRs have previously been shown to facilitate the infection of viruses like Influenza virus [35] and Respiratory Syncytial Virus [36] through interaction with their envelope proteins, this mechanism does not involve viral attachment. Influenza virus utilizes BCR signaling elicited by its envelope protein to sensitize the cell for infection, whereas RSV utilizes BCR signaling to increase the expression of its cognate receptor, CX3CR1.

*Figures:*

*Fig 3B, no axis labels.*

We have revised Figure 3B to include the appropriate axis labels, ensuring all experimental parameters and units are clearly defined.

*Fig 5A,B: conditions “A,B,C” not clearly labeled so difficult to interpret.*

We acknowledge the lack of clarity regarding the ABC treatments in Figure 5, Panels A and B. We have revised both the figure legend and the main text to elaborate in detail on the components and procedures involved in the ABC treatments, ensuring the terminology is thoroughly defined for the reader.

Line 761-769

These infections were performed in the presence or absence of control antibodies (Block A), anti-ACE2 antibodies (Block B), or the protease inhibitor E64d (Block C). Two days post-infection, viral infection was quantified by measuring firefly luciferase activity in the lysates of the infected cells. N=3 and averages are shown with SD (error bar). This experiment was repeated twice in singlicate and once in triplicate, and the results of the triplicated experiments are shown. **B)** Infection of CD79 293T cells, with or without ectopic expression of anti-SARS-CoV-2 BCR (REGN10933 and CB6), with SARS-CoV-2 VLP carrying a firefly luciferase reporter gene in the presence and absence of control (Block A) and anti-ACE2 antibodies (Block B), or E64d (Block C).

*References:*

*Some references are duplicated (#13 and 43).*

We have removed the duplicate references (formerly #13 and #43) and ensured that the remaining citations are correctly numbered and cross-referenced throughout the revised manuscript.

Methods section:

*Should be reorganized to follow presented results. Organize to ZIKV experiments, then HIV, then SARS2 experiments.*

We have reorganized the Materials and Methods section to align with the sequence of the presented results, specifically grouping the procedures by ZIKV experiments, followed by HIV, and concluding with the SARS-CoV-2 experiments.

**REVIEWER 3**

**Major Issues**

*The authors should provide (either in the main manuscript or the supplementary information) representative primary flow cytometry data plots showing the gating strategy and supporting the derived data presented in graph form (Figures 1B/C/E, 3C/E, 4C/D). Details on the flow cytometry method, e.g., cytometer model(s) and configurations, should be provided.*

We have updated the manuscript as follows:

Primary Data and Gating Strategy: We have included representative flow cytometry plots and the corresponding gating strategies. These are now shown in Fig S1A and B, S2 A and B, and S3C and D to provide direct visual support for the derived data.

Methodological Details: The Materials and Methods section has been updated (under the "Flow Cytometry" subheading) to include specific details regarding the cytometer models used, laser configurations, and detector settings as below.

Line 462-469

Flow cytometry

Flow cytometric analysis was performed using an Attune NxT Flow Cytometer (Thermo Fisher Scientific) equipped with four lasers (405 nm, 488 nm, 561 nm, and 637 nm) and 14 detection filters. The 405 nm laser utilized 450/50, 525/50, 610/20, 660/20, 710/50, and 780/60 nm filters; the 488 nm laser used 530/30 and 695/40 nm filters; the 561 nm laser used 585/16, 620/15, and 780/60 nm filters; and the 637 nm laser used 670/15, 720/30, and 780/60 nm filters. Resulting FCS 3.1 or 3.0 data files were analyzed with FCS Express 5 or 7 ( De Novo Software, Pasadena, CA).

Major comments:

*1. The authors should more thoroughly discuss published work on alternative mechanisms of SARS-CoV-2 entry/infection. Examples that were not cited include Karthika et al (PMID 34359983) and Han et al (PMID 37967509), but there are other relevant publications*

We thank the reviewer for this insightful suggestion. We agree that discussing ACE2-independent entry pathways provides a more comprehensive overview of SARS-CoV-2 tropism.

We have now incorporated a discussion of these alternative mechanisms, including the roles of TMEM106B, ASGR1, and GRP78, and have cited the suggested publications (Han et al. and others) in the revised manuscript.

Line 350-355

Several other host factors have similarly been shown to function as alternative spike-binding receptors. TMEM106B, a lysosomal transmembrane protein enriched in the brain and gastrointestinal tract, binds the spike RBD and promotes endosomal fusion [68]. In hepatocytes, ASGR1 engages both the RBD and NTD to support infection of ACE2-low cells, while cell-surface GRP78 mediates ACE2-independent spike binding and viral entry into monocytes [69] [70].

*2. The authors should address what is known regarding the neutralizing activity of the BCRs used and how this could influence the experimental results, especially for HIV-1.*

We thank the reviewer for highlighting this important point. We have addressed the neutralizing activity and binding characteristics of the BCRs used in this study as follows:

ZIKV

Line 141-145

We engineered anti-ZIKV BCRs derived from the antibodies ZKA190 and ZKA230. While ZKA190 targets domain III of the E protein, ZKA230 recognizes quaternary epitopes present on infectious virions but absent on soluble proteins. Although both antibodies potently neutralize ZIKV in Fc receptor-negative cells, they are known to induce antibody-dependent enhancement (ADE) in cells expressing Fc receptors.

HIV-1

Line 200-204

The two gp160 CD4-binding domain-specific antibodies, 3BNC117[40] and VRC07[41], were converted into BCRs. In addition, we also converted an anti-HIV-1 antibody-like molecule eCD4-Ig, which uses the ectodomain of CD4 instead of Fab as the gp160-binding domain[42]. All three molecules neutralize HIV-1 by competitively inhibiting the interaction between host CD4 and the viral envelope.

SARS CoV2

Line 242-245

While both REGN10933 and CB6 neutralize SARS-CoV-2 by targeting the ACE2-binding site, they exhibit different conformational preferences: REGN10933 binds exclusively to the S protein in the up (open) state, whereas CB6 can engage the S protein in both the up and down (closed) states.

Regarding the relationship of neutralizing activities of BCR and infection, we have added the following sentences as below.

Line 325-328

We utilized BCRs derived from ZIKV-neutralizing antibodies. Since DC-SIGN, which binds the N-glycans of the ZIKV E protein, can facilitate viral attachment and infection, neutralizing activity may not be strictly required for BCR-mediated entry, as observed for ADE mediated by non-neutralizing antibodies against ZIKV.

Regarding the relationship between the neutralizing activity of anti-HIV-1 BCRs and HIV-1 entry, we have explained our rationale for utilizing BCRs that target the CD4-binding site of gp160. This reasoning is supported by our data on the abortive virus binding mediated by DC-SIGN, which binds to non-CD4 binding sites of gp160 (please see lines 187–199).

*3. For all experiments, the authors should provide clarity on steps to establish the reproducibility of their findings, e.g., number of replicates per experiment and number of experiments conducted. Representative experimental data should be noted as such.*

We have updated the manuscript and figure legends to ensure transparency regarding the experimental design. Specifically, all figures now indicate the number of independent experiments conducted. Furthermore, we have explicitly labeled all representative data as such in the respective figure legends.

Line 651-652

This experiment was repeated twice in singlicate and once in triplicate, and representative flow cytometry histograms are shown.

Line 662-664

This experiment was repeated twice in singlicate and once in triplicate, and the results of the triplicated experiments are shown.

Line 668-670

This experiment was repeated once in singlicate and once in triplicate, and the results of the triplicated experiments are shown.

Line 719-720

This experiment was repeated three times in singlicate and once in triplicate, and the results of the triplicated experiments are shown.

Line 731-733

This experiment was repeated twice in singlicate and once in triplicate, and the results of the triplicated experiments are shown.

Line 745-747

This experiment was repeated twice in singlicate and once in triplicate, and the results of the triplicated experiments are shown.

Line 757-758

This experiment was repeated twice in singlicate and once in triplicate, and the results of the triplicated experiments are shown.

Line 765-766

This experiment was repeated twice in singlicate and once in triplicate, and the results of the triplicated experiments are shown.

Line 771-772

This experiment was repeated twice in singlicate and once in triplicate, and the results of the triplicated experiments are shown.

*4. Did the studies of ADE cited (lines 283-284) use the same SARS-CoV-2-specific Abs and cells as the authors’ experiments? The authors should comment on why ADE did not mediate infection but binding to cell-surface immunoglobulin did. (This differs from flaviviruses.)*

We have clarified in the text that one of the antibodies used in our study, CB6, has been previously investigated in the context of ADE. Previous studies have shown that CB6-mediated ADE is typically ACE2-dependent.

While the exact mechanism remains to be fully elucidated, we hypothesize that the membrane-anchored nature of the BCR provides a structural advantage. Specifically, the BCR may orient the viral Spike (S) protein in closer proximity to the host cell membrane than a soluble antibody-Fc receptor complex would. This increased spatial proximity may lower the barrier for the fusion.

Line 341-342

Of note, CB6 was previously shown to mediate ADE in an ACE2-dependent manner [58].

Line 345-349

Several antibodies against the S protein are known to induce conformational changes in the S protein upon binding to it [62]. Complete conformational changes of the envelope protein require cleavage by proteases [63] [64]; thus, binding of antibodies alone might not be sufficient to induce complete conformational changes of the envelope protein, but their BCR form might bring S proteins proximal to the proteases.

*5. ZKA230 BCR gave higher virion binding than ZKA190 BCR (Figure 1A) but lower infection (Figure 1B). How do the authors explain this finding?*

As possible explanation for this discrepancy, we have added the following sentences below to the discussion.

Line 328-331

However, the discrepancy in the ability of two anti-ZIKV BCRs, ZKA190 and ZKA230, to mediate virus binding and infection in Ramos cells (Figure 1A and B) suggests that additional factors, such as the capacity of a BCR to prevent pH-dependent conformational changes in the E protein, influence the efficiency of BCR-mediated viral entry.

*6. Line 199-200- It is not clear why binding was considered only "slightly increased" based on Figure 3D.*

We agree with the reviewer that "slightly" is a subjective descriptor. The data presented in Figure 3D should allow readers to draw their own conclusions regarding the extent of the enhancement. To ensure an objective presentation of the results, we have removed this word from the manuscript.

*7. Lines 243-247- This text should be moved to Discussion.*

We agree that the text in Lines 243–247 was not ideally placed. However, as these sentences provide the necessary rationale for the subsequent SARS-CoV-2 replicon experiments, we believe they are essential for the logical flow of the Results section. Accordingly, we have relocated this text to the beginning of the section introducing the replicon assays (now Lines 271–275) to better transition into those experiments.

*8. Lines 360-372- Several cell lines/types listed (Raji, Daudi) do not appear to have been used in the experiments presented. A source or reference should be provided for CD79, TIM-1, and ACE2 293T cells.*

We thank the reviewer for pointing this out. We have removed the mention of cell lines (Raji and Daudi) that were not utilized in the final results of this study. Additionally, we have included the appropriate citations for the CD79, TIM-1, and ACE2 293T cells in the revised manuscript.

9. Lines 373-390- The expression of soluble Ig is not relevant to the data presented. The text mistakenly refers twice to "light-chain" sequences. The expression of BCRs as ScFv (lentiviral expression) noted here should be clarified elsewhere in the manuscript.

We thank the reviewer for these corrections. We have removed the section on soluble Ig expression and corrected the "light-chain" sequence errors. Additionally, we have clarified that BCRs were expressed as scFvs via lentiviral vectors within the figure legends and methods section for better visibility.

*10. Lines 391-406- A source or reference should be provided for plasmids pCMV4-BlaM-Vpr and Intron2 Src Scarlet.*

We thank the reviewer for noting this omission. We have now included the appropriate references for both the pCMV4-BlaM-Vpr and Intron2 Src Scarlet plasmids in the revised manuscript (see References 45, Line 456).

*11. Line 462- "ZIM-V" is an error.*

ZIM-V" has been corrected to "AIM-V" in the revised manuscript.

*12. Line 479- Clarify "1LFlag1L".*

We have clarified the term "1LFlag1L" in the revised manuscript (Lines 511–512, 691-692). We now explicitly define it as a modified Sindbis virus envelope and have included the appropriate reference [4] to support its use for targeted *in vivo* B-cell transduction.

*13. Line 482- Text refers to splenocytes isolated "as described above" but there is no relevant text.*

We have now included a detailed description of the splenocyte isolation procedure in the Materials and Methods section.

Line 515-516

Spleen cell suspensions were prepared by passing spleen tissue through a 40 µm cell strainer, followed by red blood cell lysis using ACK lysis buffer.

*14. Lines 505-506- Text is duplicated.*

The duplicated text has been removed in the revised manuscript.

*15. Line 508- "5" in "105" should be an exponent.*

The formatting has been corrected, and "5" is now properly presented as an exponent (10^5^) in the revised manuscript.

*16. Line 519- Details for heat inactivation should be included.*

We have updated the manuscript to include the specific parameters for heat inactivation. The text now specifies that the virus was inactivated at 60°C for 30 minutes (Line 522).

*17. Line 526- "previously described" needs a citation.*

We have added the appropriate citation for the described method (Reference37) at Line 595 of the revised manuscript.

*18. Figure 1D- The additional control of IgG1 staining on non-transduced (FLAG-negative) IgD+ B cells should be provided.*

As requested, we have included the flow cytometry data for IgG1 staining on non-transduced (FLAG-negative) IgD+ B cells in the revised Figure 1D.

*19. Figure 3A- the black and red curves should be labeled in the legend.*

We have updated the figure legend for Figure 3A to clearly define the curves. The legend now includes the following description:

Line 709-711

The black line represents staining with an isotype control antibody, and the red curve represents staining with antibodies against the indicated antigens (CXCR4 or CD4).

*20. Figure 3C, E- The legend should indicate the method for detection of infected cells.*

The figure legends for Figures 3C and 3E have been updated to include the detection method. The following description was added:

Line 716-718 and 726-728

Two days after infection, infected cells were identified via intracellular staining using a PE-conjugated antibody against the HIV-1 Gag protein, followed by flow cytometric analysis.

*21. Figure 4A- Why are data provided for 10 mcg dose only for TIM-1-transduced cells?*

While TIM-1 can facilitate SARS-CoV-2 binding, it is significantly less efficient than ACE2. Consequently, a higher viral input was required to achieve comparable levels of binding in TIM-1-transduced cells, though this increased titer still does not support viral entry to the same extent as ACE2. We have clarified this in the Results section as follows:

Line 249-253

While TIM-1 facilitates SARS-CoV-2 attachment, it is significantly less efficient than ACE2. A higher viral titer was required to achieve binding levels on TIM-1-expressing cells comparable to those on ACE2-expressing cells (Fig. 4A). However, even with this increased input, TIM-1 failed to support viral entry at levels equivalent to ACE2 (Figs. 4B and C), a finding consistent with previous reports by the Maury group [44].

*22. Figure 4C, D- These panels are mislabeled in the figure. The method for determination of transduction should be explained in the legend.*

We have rectified the mislabeling in Figures 4C and 4D. Additionally, the figure legend has been updated to include the method used to determine transduction efficiency:

Line 750-751

Transduction was quantified by analyzing EGFP transgene expression using flow cytometry.

*23. Figure 5A, B- "Block" A, B, and C require explanation in the legend.*

We have revised both the figure legend and the main text to elaborate in detail on the components and procedures involved in the ABC treatments, ensuring the terminology is thoroughly defined for the reader.

Line 761-763

These infections were performed in the presence or absence of control antibodies (Block A), anti-ACE2 antibodies (Block B), or the protease inhibitor E64d (Block C).

Line 766-770

Infection of CD79 293T cells, with or without ectopic expression of anti-SARS-CoV-2 BCR (REGN10933 and CB6), with SARS-CoV-2 VLP in the presence and absence of control (Block A) and anti-ACE2 antibodies (Block B), or E64d (Block C). N=3 and averages are shown with SD (error bar).

*24. Figure S1A- Why was gating so inconsistent in this experiment? The authors should include negative control staining (no virus added) for all cell lines.*

We apologize for the inconsistent gating in the original Figure S1A, which was due to a software error. We have corrected the figure using a consistent gating strategy across all samples. Additionally, we have added the requested negative controls (no virus) for all cell lines to the revised figure to ensure clear comparisons
