# Supplementary material for: B Cell Receptor’s function in virus entry: Anti-SARS-CoV-2 B cell receptors can mediate viral entry in an ACE2-independent mechanism
Source: PLoS Pathog. 2026 Feb 6;22(2):e1013946. doi: 10.1371/journal.ppat.1013946 (PMC12893656; doi:10.1371/journal.ppat.1013946)
Supplement: Attachment — Submitted filename: 012226 Responses to reviewers.docx [file ppat.1013946.s007.docx]

**Responses to reviewers' comments**
Italic font indicates the comments of the reviewers, and blue regular font for author responses.

**RESPONSE TO SPECIFIC COMMENTS**

**Journal Requirements**

*1) Please ensure that the funders and grant numbers match between the Financial Disclosure field and the Funding Information tab in your submission form. Note that the funders must be provided in the same order in both places as well.*

We have verified that the funders and grant numbers match across the Financial Disclosure field and the Funding Information tab. We have also ensured that the funders are listed in the same order in both sections.

**Reviewers' Comments:**

**Part I - Summary**

*Reviewer #1: The authors have addressed my concerns with the manuscript*

*However, I noticed in Figure 5c and d, that the right hand Y axis are mislabeled. They should read* *N2 copy #/GAPDH.*

Thank you for pointing out this labeling error. We have corrected the right-hand Y-axis in Figures 5c and 5d to read 'N2 copy #/GAPDH' as suggested.

*Reviewer #3: Although the revised manuscript addresses many of my concerns, there are two major issues that should be addressed further, one pertinent to evaluating the technical quality of the work and one pertinent to providing an appropriate summary of prior work.*

We have revised the Figure Legends and the Materials and Methods section to clarify the technical aspects of our study. Furthermore, we have expanded the Discussion to better incorporate and synthesize relevant prior work. Please see our specific responses to the major issues below.

**Part II – Major Issues: Key Experiments Required for Acceptance**

*Reviewer #3:*

*1. In the prior review, it was suggested that the authors "more thoroughly discuss published work on alternative mechanisms of SARS-CoV-2 entry/infection". In response, the authors cited additional prior studies as suggested. However, this text (lines 350-355) is offered in isolation while the authors retain other text stating that SARS-CoV-2 infection is dependent on ACE2 (e.g., lines 48, 227, 336). The other literature offers additional evidence of ACE2-independent infection that should be integrated into the interpretation of the results presented here.*

We agree that these prior works were not well integrated into our results. We have now modified our Discussion to better incorporate the implications of these prior studies into our findings, as detailed below.

Line 350-358

Recent studies have identified several host factors that function as alternative spike-binding receptors [66] [67] [68]. For instance, TMEM106B, a lysosomal protein enriched in the brain and GI tract, binds the spike RBD to promote endosomal fusion[66]. Similarly, ASGR1 supports infection in hepatocytes; notably, while ASGR1 binds both the RBD and the N-terminal domain (NTD), only RBD-binding mediates ACE2-independent entry [67]. These findings suggest that the specific binding site of an alternative receptor is a critical determinant for inducing the necessary conformational changes in the S protein. We have demonstrated BCR-dependent viral entry via RBD binding, and it will be informative to further investigate the molecular mechanisms of BCR-mediated entry using BCRs that bind to domains other than the RBD.

*2. In the prior review, it was suggested that the authors provide additional primary flow cytometry data and clarity on steps to establish the reproducibility of their findings. The authors have addressed these points with substantial detail. Figure S2 provides the primary flow cytometry data showing infection of cells by HIV-1 based on detection of intracellular Gag p24 with PE-conjugated antibody. However, the x axis on the flow cytometry plots is labeled “eGFP”; this needs to be reviewed and the inconsistency corrected. (Panel B in the figure is also mislabeled as panel E in the legend.)*

We thank the reviewer for identifying these inconsistencies. We have corrected the x-axis label in Figure S2 from 'eGFP' to 'Gag p24' to accurately reflect the intracellular staining performed. Additionally, we have corrected the figure legend to ensure that Panel B is properly identified, resolving the mislabeling as Panel E. The revised figure and legend have been updated in the manuscript.

*In any case, the positive control, cells expressing CD4, showed only ~6% infection with high reproducibility across triplicate samples in this experiment, which was much lower than the % infection shown in Figure 3C using the same cell line. Both experiments were performed additional times (albeit without replicates), but the consistency of the data from the other experiments is not discussed. Importantly, these results (Figures S2B and 3E) are central to the authors’ conclusion that virus-specific BCRs are insufficient to mediate HIV-1 infection, unlike ZIKV and SARS-CoV-2.*

Thank you for pointing this out. The apparent inconsistencies between the results in Figures 3C and 3E, as well as Figures S2A and S2B, stemmed from an incomplete explanation in the Materials and Methods section. We initially confirmed the CD4 and CXCR4-dependency of HIV-1 infection through transient transfection of CD4 expression vectors into CD79 and CD79 CXCR4 293T cells. After establishing that both molecules are essential for infection, we generated a CD79CXCR4 293T cell line stably expressing CD4 to serve as the positive control for the binding and infection assays shown in Figures 3D, 3E, and S2B. We have updated the Materials and Methods section and the figure legends accordingly.

Line 529-561

**HIV-1 binding assay**

CD79 CXCR4 293T cells were generated by transducing CD79 293T cells with a lentiviral vector expressing human CXCR4. CD79 293T and CD79 CXCR4 293T cells were transiently transfected with an expression vector for human CD4 and the expression levels of CD4 and CXCR4 were analyzed by staining the cells with an APC-conjugated anti-human CD4 antibody (Biolegend, San Diego, CA) and a BV421-conjugated anti-human CXCR4 antibody (Biolegend), followed by flow cytometric analysis. To analyze the role of CD4 and CXCR4 in HIV-1 binding, CD79 293T and CD79 CXCR4 293T cells, with or without transfection of an expression vector for human CD4, DC-SIGN, a control (REGN), or an anti-HIV-1 BCR, or CD79CD4CXCR4 293T cells (5×10^5^ cells) were incubated with Scarlet-labeled HIV-1 gp160 pseudotyped lentiviral vectors (100 ng p24/ml, 200 µL) for one hour at 37°C. After washing with AIM-V, virus binding was analyzed by flow cytometry. After confirming the CD4- and CXCR4-dependency of HIV-1 infection, we sorted CD79 CXCR4 293T cells stably expressing CD4 using FACSAria III (BD Biosciences, Franklin Lakes, NJ). These cells, designated as CD79CD4CXCR4 293T, were utilized as a positive control for subsequent HIV-1 binding and infection assays. To analyze BCR-mediated HIV-1 binding, CD79 CXCR4 293T cells (with or without transfection of an expression vector for a control [REGN] or an anti-HIV-1 BCR) and CD79CD4CXCR4 293T cells (5×10^5^ cells) were incubated with the Scarlet-labeled vectors and analyzed by flow cytometry as described above.

**Infection of HIV-1**

To analyze the role of CD4 and CXCR4 in HIV-1 binding, CD79 293T and CD79 CXCR4 293T cells, with or without transfection of expression vectors for human CD4 were seeded into 48-well plates (1×10^5^ cells/0.5 ml medium/well). One day after seeding, the cells were incubated with 250 µL of infectious or heat-inactivated HIV-1NL4-3 (25 ng p24/ml) for 2 hours at 37°C. The virus was then replaced with 500 µL of fresh medium. Two days after infection, the cells were trypsinized, fixed with fixation buffer (Life Technologies), and stained with a PE-conjugated anti-HIV p24 antibody (Beckman Coulter) in permeabilization buffer (Life Technologies). Gag expression levels were then analyzed by flow cytometry. To analyze BCR-mediated HIV-1 infection, CD79 CXCR4 293T cells (with or without transfection of an expression vector for a control [REGN] or an anti-HIV-1 BCR) and CD79CD4CXCR4 293T cells were seeded into 48-well plates (1×10^5^ cells/0.5 ml medium/well). One day after seeding, the cells incubated with 250 µL of infectious or heat-inactivated HIV-1NL4-3 (25 ng p24/ml) and analyzed for infection as described above.

Line 738-749

**D)** After confirmation of CD4 and CXCR4 dependency of HIV-1 infection, we sorted CD79 CXCR4 293T cells stably expressing CD4, designated it as CD79CD4CXCR4 293T, to use it as a positive control of HIV-1 binding and infection. To evaluate the binding of Scarlet-labeled HIV-1, CD79 CXCR4 cells (with or without ectopic expression of DC-SIGN, a control BCR (REGN), or anti-HIV-1 BCRs derived from 3BNC117, VRC07, and eCD4-Ig) and CD79CD4CXCR4 293T cells “shown as CD4) were analyzed. The cells were incubated with Scarlet-labeled HIV-1 gp160 pseudotyped lentiviral vectors for one hour at 37°C. Virus binding was analyzed by flow cytometry. **E)** To evaluate HIV-1 infection, CD79-CXCR4 cells (with or without ectopic expression of DC-SIGN, a control BCR (REGN), or anti-HIV-1 BCRs derived from 3BNC117, VRC07, and eCD4-Ig) and CD79CD4CXCR4 293T cells (shown as CD4) were infected with heat-inactivated (HI) or infectious (I) HIV-1NL4-3.

Line 1165-1169

**B)** Representative flow cytometric profiles of intracellular HIV-1 Gag p24 expression in CD79 CXCR4 cells with or without ectopic expression of DC-SIGN, control BCR, or anti-HIV-1 BCR derived from 3BNC117, VRC07, and eCD4-Ig, and CD79CD4CXCR4 293T cells (shown as CD4) following infection with either heat-inactivated or infectious HIV-1 NL4-3.
